# Selenium-Biofortified Alfalfa Hay Supplemented to Jersey and Holstein Dairy Heifers During the Peripartum Period: Effects on Dams and Their Offspring

**DOI:** 10.3390/ani15131866

**Published:** 2025-06-24

**Authors:** Shana Jaaf, Matteo Mezzetti, Sebastiano Busato, Erminio Trevisi, Massimo Bionaz

**Affiliations:** 1 Animal and Rangeland Sciences, Oregon State University, Withycombe Hall, Corvallis, OR 97331, USA; shana802001@yahoo.com (S.J.); sebastianobusato@gmail.com (S.B.); 2 Department of Animal Sciences, Food and Nutrition (DIANA), Università Cattolica del Sacro Cuore, Via Emilia Parmense, 84, 29122 Piacenza, Italy; matteo.mezzetti@unicatt.it (M.M.); erminio.trevisi@unicatt.it (E.T.)

**Keywords:** peripartum, immune status, oxidative stress, calves

## Abstract

The objective of the study was to assess whether supplementing dairy heifers with Se-biofortified hay during the last 40 days of pregnancy and early lactation improves performance, metabolism, oxidative status, and immune response in the dams and their calves. Ten Jersey and eight Holstein pregnant dairy heifers were divided into two groups and supplemented with Se-biofortified or a control alfalfa hay from 40 days before to 2 weeks postpartum. The calves born to the heifers were also monitored during the first month of life. Supplementation of Se-biofortified hay improved milk yield in Holstein heifers only, but had minor effects on blood parameters measured and the liver transcriptome. Similarly, we did not detect any impact of feeding Se-biofortified hay to heifers on their offspring. The data indicated a better immune system and oxidative status in Holstein compared to Jersey heifers. Overall, our data did not support the hypothesis that supplementing Se-biofortified hay to pregnant heifers improves their performance and the health of dams and offspring, at least at the dose we provided.

## Simple Summary

The period from pregnancy to lactation and the early phase of life after calving are the most challenging for dairy cows and calves, respectively, due among others to heightened inflammation, dysfunction of the immune system, and oxidative stress that can negatively affect their offspring, Selenium, especially if fed in an organic form, as in the case of biofortified hay, can aid in improving the immune system decreasing inflammation and oxidative stress. To assess this, we fed selenium biofortified alfalfa hay to pregnant dairy Holstein and Jersey heifers and evaluated their performance and, through the analysis of several blood parameters, their physiological status as well as the status of their offspring. Our data indicated that feeding selenium-biofortified alfalfa hay to pregnant Holstein or Jersey heifers had minimal effects on the performance and health status of the dairy cows and their offspring.

## 1. Introduction

The transition from the dry period to calving and the perinatal phase are widely documented as the most critical periods in the life of a dairy cow [1,2]. Transition cows undergo complex physiological and metabolic changes [3], often experiencing immune dysfunctions and oxidative stress [4]. A large amount of circulating non-esterified fatty acids (**NEFA**), typical of early postpartum cows, can contribute to increased production of reactive oxygen species (**ROS**) when these fatty acids enter hepatocytes and are converted to energy in the mitochondria [3,5,6]. ROS are produced not only by the NEFA catabolism but also by active phagocytes [7]. An imbalance between high ROS production and low antioxidant defenses generates oxidative stress [8]. Additionally, early postpartum dairy cows experience inflammatory-like conditions that can impair the ability of the liver to cope with the significant metabolic and immune challenges that follow [9,10]. These combined factors contribute to an increased incidence of diseases [11,12].

The metabolic and oxidative stress experienced by dairy cows during the transition period negatively impacts the immune system [13]. However, immune dysfunction in the early postpartum period in dairy cows is also strongly influenced by nutritional deficiencies, as demonstrated by the positive effects of various supplements on the immune system during this stage of lactation [14,15]. Besides dairy cows, the immune system is crucial for neonatal calves. Proper immune function in calves largely depends on colostrum-mediated passive immune transfer and the efficient functioning of innate immune defenses necessary to survive [16].

Among trace minerals, selenium (**Se**) enhances the antioxidative status and the innate and adaptive immune response [17,18,19]. Supplementation with Se can also modulate the animals’ inflammatory response and metabolic activity [17]. Se is a cofactor in the glutathione peroxidase (**GPx**) enzymatic system [18,19,20,21,22]. Therefore, maintaining adequate Se levels in the body is essential for sustaining redox balance and supporting a healthy immune system. Thus, Se deficiencies could detrimentally affect the adaptation of both dams and their calves by lowering the efficiency of body antioxidants and negatively impacting the immune system, possibly compromising the resilience of the animals to diseases [23,24].

Several regions, including the Northwestern US, have low levels of Se in the soil, which results in forages with low Se concentrations [25]. Thus, cows raised in these areas do not receive adequate Se from forages, and supplementation is required. As inorganic Se sources (i.e., selenate and selenite) have a low acute toxicity threshold in cattle, a maximum of 3 mg/head per day is recommended in dairy rations [26], and a limit of 0.3 mg/kg dry matter (ppm) has been set by the Food and Drug Administration (FDA) to include this trace mineral in feed additives. Compared to inorganic Se, organic Se is less toxic and can be used as a safe supplement to match the requirements of dairy cows [27,28]. Agronomic biofortification of forages can be an excellent strategy to supplement organic Se to dairy cows raised in areas with low Se in the soil [29,30].

In our previous study [31], feeding 1 kg DM/100 kg body weight (**BW**) of Se-biofortified alfalfa hay to pregnant dairy heifers starting 40 days before parturition through 14 days in milk improved the Se status and the GPx activity in whole blood of cows and calves. However, the effect of Se-biofortified alfalfa on the metabolic adaptation of the cows to the peripartum phase and the adaptation of their newborn calves to the perinatal period was not assessed.

Based on previous findings on the effects of organic selenium in dairy cows, we hypothesized that supplementing cows with a relatively small amount of Se-biofortified alfalfa hay during the dry period and early lactation would enhance their performance, metabolism, oxidative and immune status—as well as those of their newborn calves.

## 2. Materials and Methods

### 2.1. Animal Management and Experimental Design

The study was conducted at the Oregon State University Dairy Research Center (Corvallis, OR) from September 2017 to May 2018. The overall experimental design is shown in Supplementary File S1, Figure S1. Ten Jersey (Jer) and eight Holstein (Hol) pregnant heifers were enrolled in the study 45 d before expected parturition, equipped with a pedometer (Afikim, Israel), moved into a pen fitted with Calan gates (American Calan, Northwood, NH), and fed ad libitum with total mixed ration (**TMR**) twice daily. At approximately 40 days from calving (**DFC**; −38 ± 5 days from the actual calving date), the heifers were blocked by breed and were randomly assigned to two groups, both receiving 1 kg DM/100 kg BW of supplemental alfalfa hay, chopped and carefully mixed by hand into the daily TMR until 14 DFC, but with the **Sel** group (5 Jer and 4 Hol) receiving a Se-biofortified alfalfa hay containing 3.25 mg Se/kg DM, whereas the **Ctr** group (5 Jer and 4 Hol) received standard alfalfa hay containing 0.43 mg Se/kg DM. After calving, the cows were milked twice daily at 0500 and 1800 h, and at 15 DFC, they were moved to the herd and fed the standard TMR diet for lactating cows.

Three different batches of alfalfa hay from a prior experiment [32] were used for the present study. Two alfalfa hay batches obtained from fields fertilized with 45 and 90 g sodium selenite/ha were mixed in equal amounts to obtain alfalfa with 3.25 mg Se/kg DM and used for the treatment group. We used alfalfa hay containing 0.43 mg Se/kg DM as a control obtained from a field not fertilized with sodium selenite. Chemical analysis results of the three hays, obtained from a commercial laboratory (Dairy One Forage Testing Laboratory, Ithaca, NY, USA), are reported in Supplementary File S1, Table S1. During the dry and lactation period, the heifers received ad libitum TMR formulated for dry and lactating cows, respectively. TMR was provided twice daily, approximately at 07:30 AM and 04:30 PM. Before feeding, TMR was mixed by hand with the chopped Se-biofortified alfalfa hay or the control alfalfa hay for each animal. Samples of TMR were collected once a month during the trial and preserved at −20 °C until analysis. Except for Se, the chemical analysis of the TMR was performed by a commercial laboratory (Dairy One Forage Testing Laboratory, Ithaca, NY, USA). The composition and chemical analysis of the TMRs, including the analysis for Se, are reported in Supplementary File S1, Table S2. The cows received a commercial Trace Mineral Salt Brick (cat#270220, American Stockman, Overland Park, KS, USA) without Se, which was inserted into the Calan gate using a commercial plastic adaptor. The animals were milked twice a day, at 04:15 AM and 2:15 PM, and milk yield was recorded by the Afilab system (Afimilk, Kibbutz Afikim, Israel). The heifers were monitored daily for health status, dry matter intake, and milk yield, and weekly for BW. The TMR contained the NRC-recommended levels of Se (Supplementary File S1, Table S2). Thus, none of the animals were Se-deficient, as confirmed by the level of Se in whole blood [31].

All calves born from the cows enrolled in the experiment were immediately moved to individual Calf-Tel Pen System Calf hutches (122 cm W × 182 cm L × 114 cm H) bedded with wheat straw and received 3 L of colostrum from their mothers within 6 h after birth and 2 L of colostrum for their second feeding 12 h after birth. Thereafter, the calves were fed raw cow milk collected from the bulk tank at 0700 h, with ad libitum access to a starter concentrate and water for the entire experimental period. The calves were considered in the two homogeneous groups according to the treatment received by their mothers (i.e., **Sel** and **Ctr**). The Sel group calves comprised 5 female Jerseys, 3 male and 1 female Holstein, while the Ctr group calves comprised 1 male and 4 female Jerseys, and 1 male and 3 female Holsteins.

### 2.2. Animal Measurements, Sample Collection, and Handling Procedures

During the study, the health status of the cows and calves was monitored daily, and periodical sample and data collections were performed from −38 through 14 DFC for the cows and from 1 to 24 DFC for the calves (Supplementary File S1, Figure S1).

#### 2.2.1. Intake, Milk, and Activity

From −38 to 14 DFC, the dry matter intake (**DMI**) of the cows was recorded daily by measuring the feed provided and the residuals, and the cow’s BW was measured once a week using a walking-in scale located at the milking parlor exit and registered by the Afimilk system. The body condition score (**BCS**) of the cows was measured by three different people using a 1 to 5 scale [33]. The final BCS was calculated as the geometric mean of the three operators. Milk yield, milk conductivity, and animal activity were recorded from the cows at each milking by the Afilab system (Afimilk, Kibbutz Afikim, Israel), and data were expressed as daily values. A colostrum sample was collected within one hour of calving and immediately frozen at −20 °C to assess the immunoglobulin content. Milk samples were collected during the morning milking in tubes containing bronopol, stored at 4 °C, and sent to the Willamette DHIA laboratory (Salem, OR, USA) for assessing milk composition and somatic cell count (**SCC**). Additional milk samples were collected at 4 and 14 DFC and used to determine the milk fatty acids (**FA**s).

#### 2.2.2. Blood Samples

Blood samples were collected before the morning feeding from the jugular vein into evacuated tubes containing Na-heparin (Cat# 366480, 10 mL; Becton Dickinson, Franklin Lakes, NJ, USA). Plasma was obtained by centrifugation at 2000× *g* for 15 min at room temperature and frozen at −20 °C. Additional blood samples were collected from the cows at −10, 4, and 14 DFC and the calves at 2 and 22 DFC using K-EDTA evacuated tubes (BD Vacutainer, Becton, Dickinson and Co., Franklin Lakes, NJ, USA) for leukocyte counts, differential, and phagocytosis.

#### 2.2.3. Liver Biopsy

A liver biopsy was performed for transcriptomic analysis on the 10 Jersey cows (5 Ctr, 5 Sel). Liver samples were collected once prepartum (−10 DFC) and once postpartum (10 DFC). Following intravenous 10 mg xylazine sterile solution (20 mg/mL) (Cat# 4811, Akorn, Inc., Decatur, IL, USA), the area selected for puncture was infiltratated with a local anesthetic (lidocaine 2%, Cat# 002468, VetUS, Hanover, MD, USA) and shaved and decontaminated using povidone-iodine medical scrub (055478, Covetrus, Dublin, OH, USA), followed by a solution of 75% ethanol, applied with surgical gauze (100-1444, Henry Schein, Melville, NY, USA). A small incision was made using a #10 surgical blade (327-1504, Integra Miltex, York, PA, USA), and a trocar was used to collect liver tissue (approximately 500–800 mg). The tissue was collected on a sterile tissue culture dish (351029, Corning Falcon, Corning, NY, USA) and rinsed immediately in sterile phosphate-buffered saline (25-508P, Genesee Scientific, El Cajon,, CA, USA). The rinsed tissue was then immediately transferred to a 2 mL screwcap cryovial (89094-804, VWR, Radnor, PA, USA) filled with 600 µL of TRIzol reagent (15596026, Thermo Scientific, Waltham, MA, USA), along with two 3.2 mm beads, and subsequently snap-frozen in liquid nitrogen. All liver samples were kept at −80 °C until RNA extraction.

### 2.3. Analytical Procedures

#### 2.3.1. Plasma Profile

An aliquot of the plasma was sent to the Department of Animal Sciences, Food and Nutrition (DIANA), Università Cattolica del Sacro Cuore, Piacenza, Italy, for analysis using a clinical autoanalyzer (ILAB Taurus, Instrumentation Laboratory, Bedford, MA, USA). The concentration in plasma of glucose, NEFA, BHB, urea, creatinine, calcium, magnesium, total protein, haptoglobin, ceruloplasmin, albumin, total bilirubin, and cholesterol and the activity of aspartate aminotransferase–glutamate oxaloacetate transaminase (**AST/GOT**), gamma-glutamyl transferase (**GGT**), and alkaline phosphatase (**ALP**) were determined as previously described [34]. Following previously developed protocols, we also measured in plasma the concentration of reactive oxygen metabolites (**ROMt**) and ferric ion reducing antioxidant power (**FRAP**) [35], the activity of paraoxonase [36], the concentration of thiols [37], the activity of myeloperoxidase (**MPO**) [38], and the concentration of advanced oxidation of protein products (**AOPP**s).

#### 2.3.2. Leukocyte Count, Differential, and Phagocytosis

Total white blood cells (**WBC**s) were counted in whole blood from the cows and calves using the Leuko-tic blue kit (Cat#4013-0006/-0007/-0008, Bioanalytic, Umkirch, Germany). The pHrodo™ BioParticles™ Phagocytosis kit, containing green *S. aureus* bioparticles (Cat# P35382, Life Technologies, Carlsbad, CA, USA), was used to measure the phagocytosis of WBCs according to the manufacturer’s protocol. At the end of the protocol, the cells were stained with primary antibodies CAM36A (IgG anti-CD14; Cat# 6-9-03) and CH138A (IgM; neutrophils marker; Cat# 2001) from the Washington State University Monoclonal Antibody Center, Pullman WA. Allophycocyanin-conjugated goat anti-mouse IgG (Cat# M30005, Caltag Laboratories, Burlingame, CA, USA) and R-Phycoerythrin-conjugated goat anti-mouse IgM (Cat# 662587, Invitrogen, Waltham, MA, USA) were used as secondary antibodies. The samples were centrifuged at 350× *g* for 5 min at 20 °C, and the pellet was resuspended in 200 µL of the appropriate primary antibody solution and incubated for 1 h on ice. After incubation, the samples were centrifuged at 1000× *g* for 5 min at 20 °C, and the pellet was resuspended in 200 µL of secondary antibodies solution (1:200) and incubated for 30 min on ice. After incubation, the samples were centrifuged at 1000× *g* for 5 min at 20 °C, and the pellet was resuspended in 1 mL of 4% formalin (Cat# 170191, Fisher Chemicals, Pittsburgh, PA, USA) + 2‰ DAPI solution and incubated for 30 min at room temperature. Finally, all the samples were centrifuged at 1000× *g* for 5 min at 20 °C, resuspended in 500 µL of PBS, filtered using a cheesecloth, and loaded into a 96-well flat-bottom plate (Cat#655 180, Cellstar, Carrolton, TX, USA) for flow cytometry assay using a Beckman Coulter CytoFLEX. Flow cytometer data for phagocytosis and differential were analyzed as previously described [39].

#### 2.3.3. Plasma and Colostrum Immunoglobulins and Milk Fatty Acids

Using previously described protocols, we measured immunoglobulin A (IgA) and immunoglobulin G (IgG) in colostrum and calf plasma samples [22] and performed milk fatty acid profiling [40] using behenic acid as the internal standard (100 μL of a 0.2 mg/mL solution, Cat#1161, Matreya, LLC, State College, PA, USA).

#### 2.3.4. RNA Sequencing

Liver samples were processed using a bead beater (Bullet Blender, Next Advance, Troy, NY, USA) at speed 6 for one minute at a time, with the tubes resting on ice between sessions, until visual confirmation of complete tissue disruption. RNA was extracted using Trizol and RNAeasy Mini Kit (74104, QIAGEN, Valencia, CA, USA) [39]. RNA Concentration and 260/230 and 260/280 ratios were determined using a SpectraDrop Micro-Volume Microplate in a SpectraMax plus 384 spectrophotometer (89212-396, Molecular Devices, San Jose, CA, USA). RNA integrity number (RIN) was assessed by the Center for Quantitative Life Sciences (**CQLS**; previously Center for Genome Research and Bioinformatics) at Oregon State University using an Agilent Bioanalyzer 2100 (G2939BA, Agilent, Santa Clara, CA, USA). The average RIN was 8.0 ± 0.6, with the lowest sample having a RIN of 6.4.

The CQLS performed high-throughput sequencing of the RNA samples using a QuantSeq 3′ mRNA-Seq Library Prep Kit FWD for Illumina (015.96, Lexogen, Greenland, NH, USA) followed by sequencing using an Illumina HiSeq3000 platform in two lanes, generating single-end reads of 100 bases in length; all 30 samples were repeated on each lane, generating two distinct sets of read outputs per sample.

Quality control was assayed using MultiQC v1.8 [41]. All read files were trimmed based on PHRED score and the presence of the adapter using BBDukuk from the BBMap suite. All read files were then re-assayed through MultiQC to verify improvement in read quality. A genome index was generated using the ARS-UCD1.2 Bos taurus genome and the ARSUCD1.2.104 annotation, using the genomeGenerate function of STAR v2.7.1 [42] (https://github.com/alexdobin/STAR, accessed on 14 May 2025). Trimmed reads were aligned against the reference genomic index using STAR, and the average overall alignment rate was >95%. For each sample, two bam files were generated; they were merged using the “merge” function of samtools v1.0 [43], and gene count matrices were generated using stringtie v 2.0. Differential expressions were determined using DeSeq2 v1.30.1 in R v4.0. Counts were filtered so that genes with four or fewer counts in five or more samples were removed. Sample clustering was visualized using principal component analysis plots. Three comparisons were generated: Ctr vs. Sel at prepartum and postpartum, and prepartum vs. postpartum. Using the Benjamini–Hochberg procedure, the *p*-values were adjusted by false discovery rate (**FDR**) using DeSeq2. FDR-adjusted *p*-values below 0.3 were considered differentially expressed genes (**DEGs**).

For the bioinformatics analysis, we used the Database for Annotation, Visualization, and Integrated Discovery (**DAVID**) [44] and the Dynamic Impact Approach (**DIA**) [45]. For both tools, the background used was the annotated count-filtered transcriptome.

#### 2.3.5. Calculations

The DMI per kg of body weight (DMI/Kg BW) was calculated as the ratio between daily DMI and weekly BW value. As previously described, energy-corrected milk (ECM) was calculated based on daily yield and butterfat, protein, and lactose contents [46]. Using the plasma parameters, we estimated the level of globulin as the difference between total protein and albumin, free calcium [47], urea-to-creatinine ratio [48], NEFA-to-albumin ratio [49], albumin-to-globulin ratio [50], and ROMt-to-FRAP ratio [51]. The proportional abundance of lymphocytes and the sum of neutrophils and monocytes in whole blood were calculated based on flow cytometer outputs.

### 2.4. Statistical Analysis

Potential outliers were examined using the PROC REG of SAS (SAS Institute Inc., Cary, NC, USA). Data with studentized t residuals ≥ 3.0 were removed prior to analyses. Missing values or values removed as outliers were replaced with the means of the treatment (**TRT**) for each breed and time point using the AVERAGEIFS function in MS Excel. The final data were analyzed in SAS software, version 9.4 (SAS Inst. Inc., Cary, NC, USA), and presented in graphs and tables as the least squares mean and pooled standard error.

Data underwent ANOVA testing using a mixed model for repeated measures (Glimmix Procedure, SAS Inst. Inc.) with the statistical model including the fixed effect of treatment (**TRT**; Ctr and Sel groups), breed (Hol and Jer), time, and their interactions, and the random effect of the individual cow.

*yijkl* = *µ* + *αi* + *δij* + *εkj* + *τl* + (*αβ*)*ik* + (*ατ*)*il* + (*αβτ*)*ikl* + *eijkl*

where *yijkl* is the response at time *l* on animal *j* of breed *k*, in TRT *i; µ* is the overall mean; *αi* is the fixed effect of TRT *i*; *δij* is a random effect of animal *j* in TRT *i*; *εkj* is a random effect of animal *j* in breed *k*; *τl* is the fixed effect of time *l*; (*αβ*)*ik* is a fixed first-order interaction effect of TRT *i* with Breed *k*; (*ατ*)*il* is a fixed first-order interaction effect of TRT *i* with time *l*; (*αβτ*)*ikl* is a fixed second-order interaction effect of TRT *i*, time *l*, and breed *k*; and *eijkl* is the random error at time *l* on animal *j* in TRT *i* with breed *k*. For the calves, the fixed effect of sex (M and F) was included in the model. The proportion of males and females was not balanced, and at 24 DFC, we did not have data from the Ctr Holstein male calf; thus, to account for the above, we used sex as a covariate in the model for all the data obtained from the calves.

The analyses used four covariance structures: autoregressive, unstructured, antedependence, and spatial power, with their heterogeneous counterparts. These were ranked according to their Akaike information criterion, with the one having the lowest criterion being chosen for final statistical analysis [51]. The data presenting a baseline underwent a covariate adjustment by adding the baseline as a fixed effect in the model. For the breed and breed × time, the covariate at baseline was not performed, but the baseline was used as data in the model, as the breed is a factor preceding any treatments.

*yijkl* = *µ* + *COV* + *αi* + *δij* + *βk* + *εkj* + *τl* + (*αβ*)*ik* + (*αβτ*)*ikl* + *eijkl*

where *yijkl* is the response at time *l* on animal *j*, accounting for the *COV* (covariate at baseline, when present) of breed *k* in TRT *i; µ* is the overall mean; αi is a fixed effect of TRT *i*; *δij* is a random effect of animal *j* in TRT *i*; *βk* is a fixed effect of breed *k*; *εkj* is a random effect of animal *j* in breed *k*; (*αβ*)*ik* is a fixed first-order interaction effect of TRT *i* with breed *k*; and (*αβτ*)*ikl* is a fixed second-order interaction effect of TRT *i*, time *l*, and breed *k*.

Significance was declared with *p* ≤ 0.05, and differences with *p* ≤ 0.10 were discussed in the context of tendencies.

## 3. Results

Most of the parameters assessed in this experiment were significantly affected by time. The time effect is not be presented in the Results section or discussed thereafter.

### 3.1. Performance, Milk Composition, and Activity of the Cows

**Figure 1:**
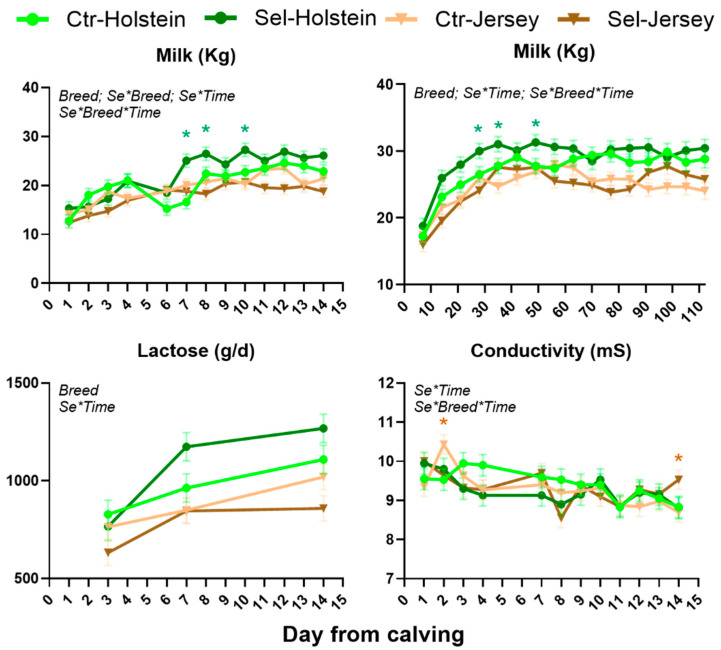
Performance and milk quality affected by interactions in Se-biofortified hay (Sel) or control hay (Ctr) Holstein or Jersey pregnant heifers from −40 through 14 days from calving. Indicated are the significant (*p* < 0.05) interactions. Significant (*p* ≤ 0.05) differences between the two diets in the same breed at the same time point are marked with * (green) for Holstein and * (brown) for Jersey.

None of the performance data were significantly affected by feeding Se-biofortified alfalfa hay (Table 1). The milk yield during the first 2 weeks postpartum and DMI were affected by the Se × Breed interaction, with higher milk yield in Holstein cows receiving Se-biofortified hay vs. Ctr. An interaction was observed for Se × Time for lactose yield due to a numerically larger yield during the first week of lactation in Holstein cows receiving Se-biofortified hay. An interaction with Se × Breed × Time was observed for milk yield during the first 2 weeks and the first 110 days postpartum, due to higher milk production in Holstein cows receiving Se-biofortified hay, while no effect was observed in Jersey cows (Figure 1). Also, conductivity was affected by the full interaction due to feeding Se-biofortified hay at 2 and 14 days postpartum, but only in Jersey cows (Figure 1).

Feeding Se-biofortified hay had a minor effect on the FA profiling of the milk. Only the proportion of the peaks corresponding to the sum of trans13 and trans14 C18:1 was significantly increased by Se-biofortified hay (Supplementary File S1, Table S3). The Δ9 desaturation activity toward C14:0 and the proportion of C20:3n3 showed a significant Se × Time interaction due to a decrease from 7 to 14 DIM in Sel but an increase in Ctr cows. The proportion of conjugated linolenic acid (C18:3CLN) was affected by a full Se × Breed × Time interaction due to a larger value at 7 DIM in Jersey cows receiving Se-biofortified hay compared to Ctr.

As expected, most performance parameters differed between the two breeds, including DMI, which had an interesting pattern early postpartum (Supplementary File S1, Figure S1). Of interest, and somewhat novel, were the higher activity, MUN, and SCC in Jersey cows compared to Holstein heifers. The FA profile of milk also differed between the breeds, particularly in the bacteria-derived fatty acids that were more abundant in the milk of Holstein vs. Jersey cows, as was the Δ9 desaturase activity toward C14:0. Differences were also detected in the proportional abundance of some of the 18:1 isomers (Supplementary File S1, Table S3).

### 3.2. Plasma Metabolic Profile of the Cows

**Figure 2:**
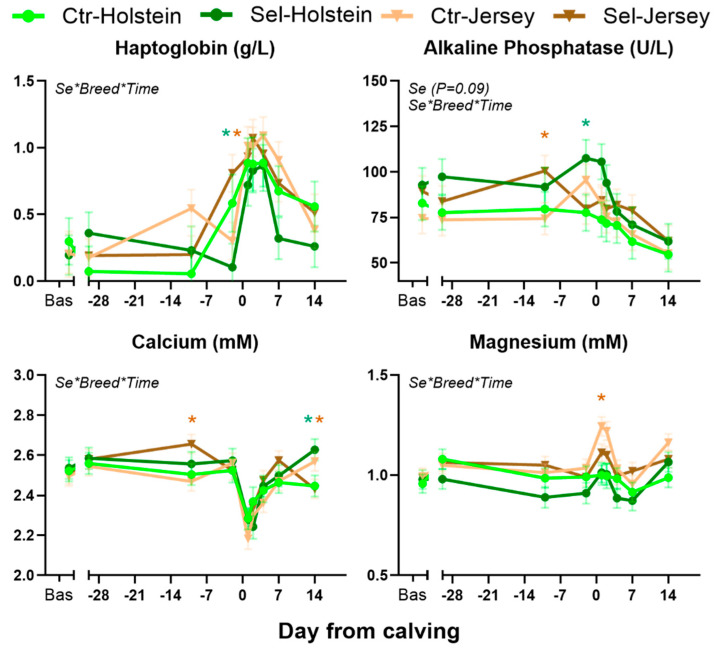
Blood parameters affected by the various interactions when Holstein or Jersey pregnant heifers were fed Se-biofortified hay (Sel) or control hay (Ctr) from −40 through 14 days from calving. Indicated are the significant (*p* < 0.05) interactions. Significant (*p* ≤ 0.05) differences between the two diets in the same breed are marked with * (green) for Holstein and * (brown) for Jersey.

Table 2 reports all the parameters measured in plasma. Figure 2 shows the temporal pattern of parameters with a significant interaction effect.

*Metabolic parameters*. None of the measured parameters related to metabolism were significantly affected by feeding Se-biofortified hay to peripartum heifers.

*Inflammatory parameters*. Ceruloplasmin was increased by feeding Se-biofortified hay to both Holstein and Jersey cows. Haptoglobin was affected by the full interaction due to a faster increase in Jersey cows fed Se-biofortified hay before parturition, while the value remained low in Holstein cows fed Se-biofortified hay. The latter also had a quick drop in the parameter during the first week postpartum. The concentration of total proteins was affected by the Se × Breed interaction, due to a decrease in globulin in Holstein cows, while an increase was observed in Jersey cows fed Se-biofortified hay.

*Liver status*. Only the activity of ALP was affected by the Se × Breed × Time interaction, with a higher ALP in Sel vs. Ctr during the late prepartum.

*Oxidative status*. Of all parameters measured related to the oxidative status, only the plasma concentration of ROM was greater overall in Sel vs. Ctr cows.

*Minerals*. Plasma concentrations of Ca and Mg were affected by the Sel × Breed × Time interaction (Figure 2). The concentration of Ca was larger at −10 DFC but lower at 14 DFC in Sel vs. Ctr in Jersey cows. In Holsteins, compared to Ctr, cows in the Sel group had higher Ca concentration at 14 DFC. The spike of Mg early postpartum was lower in Jersey cows fed Se-biofortified compared to control hay.

*Kidney function*. The creatinine concentration was lower in cows fed Se-biofortified compared to control hay; consequently, there was a tendency (*p* = 0.08) for a higher urea–creatinine ratio in the same groups.

*Differences between breeds*. Most of the blood parameters differed between the two breeds, with higher values of hematocrit, concentration of NEFA, FRAP, AOPP, and creatinine, and activity of GGT, and lower concentrations of thiol groups, ROM, and Mg in Holstein vs. Jersey cows. The NEFA, glucose, and ROM/FRAP were affected by a Breed × Time interaction, with higher glucose and ROM/FRAP at −10 DFC and lower NEFA at −2 and 7 DFC in Jersey vs. Holstein cows (Supplementary File S1, Figure S3).

### 3.3. Leukocyte Counts, Differential, and Phagocytosis in Cows

Data on the immune cells are presented in Table 3. Feeding Se-biofortified hay had a tendency (*p* = 0.06) to decrease the number of circulating WBC. The phagocytosis of leukocytes was affected by the full interaction Se × Breed × Time, due to higher values at −10 DFC in Holstein cows fed Se-biofortified hay compared to the other groups (Supplementary File S1, Figure S4).

Holstein cows had higher phagocytosis compared to Jersey cows. The WBC count was affected by the Breed × Time interaction as the values were higher in Holstein than in Jersey cows before parturition, but with a drop in the Holstein cows at 4 DFC (Supplementary File S1, Figure S4).

### 3.4. RNA Sequencing of the Liver of Jersey Cows

Results of the RNA sequencing are available in Supplementary File S2. There were 932 DEGs from −10 to +10 DFC in the liver of Jersey heifers. The bioinformatics analysis with DAVID and DIA similarly revealed an upregulation of the translational capacity of the liver, especially involving the endoplasmic reticulum, and metabolic activity, primarily related to lipid (i.e., PPAR signaling and cholesterol metabolism), glucose (i.e., gluconeogenesis), and amino acids (especially involving Gly, Ser, Thr, Val, Leu, and Ile), but a downregulation of the clearance capacity of the liver (i.e., xenobiotic metabolism), blood coagulation (e.g., complement and coagulation cascade), and healing/differentiation (i.e., angiogenesis) (Supplementary File S3).

**Figure 3:**
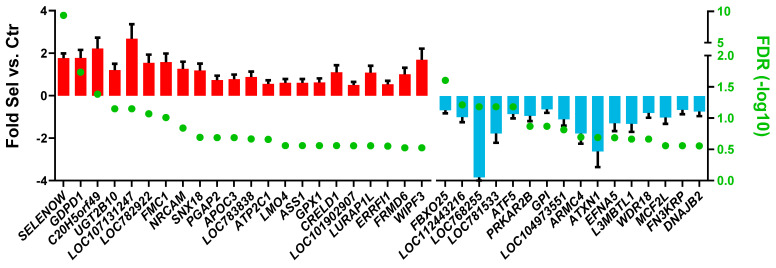
Differentially expressed genes in prepartum samples from Jersey cows supplemented with Se-biofortified alfalfa hay (Se) vs. normal alfalfa hay (Ctr). All presented transcripts have FDR-adjusted *p* < 0.3.

When evaluating the effect of feeding Se-biofortified hay, the contrast that produced the most significant number of DEGs was Sel vs. Ctr during the prepartum period, with 38 DEGs (Figure 3 and Supplementary File S2). In comparison, only 10 DEGs were detected in postpartum samples (Supplementary File S2). Among the up-regulated DEGs, two appear noteworthy: selenoprotein W (*SELENOW*) and glutathione peroxidase 1 (*GPX1*). Using the DAVID bioinformatics tool, the enrichment analysis revealed an induction of the response to selenium ion and cholesterol homeostasis and an inhibition of kinase activity and cell proliferation (Supplementary File S3). No terms were detected with DIA or in the DEGs between Sel and Ctr postpartum.

### 3.5. Blood Parameters in Calves

**Figure 4:**
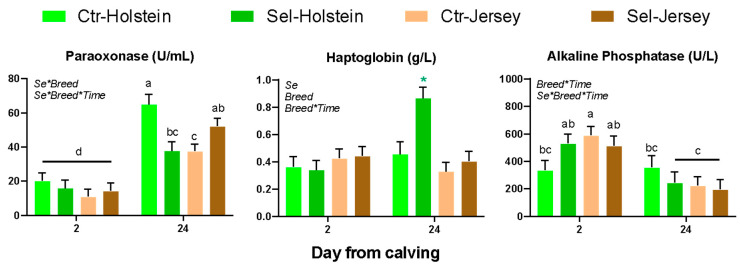
Blood parameters affected by the various interactions in calves born from Holstein or Jersey pregnant heifers fed Se-biofortified (Se) or control hay (Ctr) for the last 40 days of pregnancy. Indicated in the graphs are the significant (*p* < 0.05) interactions. Diverse letters denote statistical difference (*p* < 0.05). * denotes the group at a time point driving the Breed × Time interaction.

Among blood parameters in calves (Table 4), all except a few changed from 2 to 24 DFC. The concentrations of haptoglobin and GGT increased in the calves by feeding Se-biofortified hay to the cows. Paraoxonase was affected by a Se × Breed interaction due to larger values in Holstein calves born from dams fed the control diet at 24 DFC compared to the other groups (Figure 4). Haptoglobin increased significantly from 2 to 24 DFC only in calves born from cows fed Se-biofortified hay (Figure 4). ALP decreased from 2 to 24 DFC in all groups except in calves born from Holstein cows fed the control diet (Figure 4). The concentrations of glucose, urea, haptoglobin, and creatinine were higher in Holstein compared to Jersey calves.

### 3.6. Leukocyte Count, Differential, and Phagocytosis in Calves

Among leukocytes, feeding Se-biofortified hay to cows tended (*p* = 0.06) to decrease the circulating lymphocytes, with the highest leukocyte phagocytosis observed in Holstein calves and overall higher neutrophil phagocytosis in Holstein vs. Jersey calves (Table 5 and Supplementary File S1, Figure S5).

### 3.7. Immunoglobulins in Colostrum and Serum of Calves

The abundance of immunoglobulins in the colostrum was not affected by feeding Se-biofortified hay to dairy heifers (Table 5). There was only a tendency (*p* = 0.06) for a higher IgA in the colostrum of Holstein vs. Jersey cows. The abundance of immunoglobulins measured in the plasma of calves was not affected by the treatment of the cows; however, only IgA decreased from 4 to 24 DFC, and a full Se × Breed × Time interaction was observed for the IgG due to an increase in abundance from 4 to 24 DFC in calves from Jersey cows fed Se-biofortified hay (Supplementary File S1, Figure S6). The Holstein calves had a higher abundance of circulating IgG than the Jersey calves (Table 5). Feeding Se-biofortified hay to the dams did not affect the calves’ proportion of neutrophils and their phagocytosis capacity (Table 5).

## 4. Discussion

The animals in our study received the typical dairy farm diet, which contained an adequate level of Se. For this reason, none of the animals were deficient in Se, as confirmed by the Se level in whole blood [31]. Thus, the study should be interpreted as assessing the role of Se levels in the diet above the NRC recommendation.

### 4.1. Se-Biofortified Hay Improves Milk Yield in Holstein Cows but Does Not Affect Milk Quality

In this experiment, Holstein and Jersey pregnant dairy heifers were raised together, exposed to the same management practices, and followed across the transition to their first lactation to evaluate their responses to supplemental alfalfa hay containing different Se levels. Holstein cows had an increase in milk production when fed with Se-biofortified hay. The effect observed in Holstein cows is consistent with previous studies supplementing Se to pregnant Rambouillet ewes, mainly attributed to improved mammary gland growth, development, and vascularity [52,53]. In a recent experiment with Polypay ewes, feeding a high level of Se-yeast from 40 days before to 30 days post-lambing, we did not observe any effect on milk production [54]. The above observations are in line with prior experiments that found different adaptations to the transition period among breeds of sheep and cows, even when exposed to the same environment [54,55,56], and this could have impacted the responses to Se-biofortified hay in the two cow breeds in our experiment. As in our experiment with transition sheep [54] or transition cows [57], we did not observe any performance benefit from supplementing organic Se to Jersey dairy cows.

In cows and humans [58,59], a heightened amount of PUFAs has been documented as the main alteration in milk FA induced by Se administration, mainly attributed to the preventive effect exerted by milk GPx on the PUFAs oxidation by free radicals [60]. However, in our experiment, Se-biofortified hay did improve GPx activity in blood but did not affect milk GPx content [31]. Thus, it might not be surprising that we did not observe an increase in PUFAs by feeding Se-biofortified hay in our experiment. Similarly, in sheep supplemented with a high dose of Se-yeast, we did not observe significant changes in the fatty acid profiling of the milk, including PUFAs [54].

### 4.2. The Immune System in Cows Is Not Significantly Affected by Feeding Se-Biofortified Hay

Supplementation of Se has been associated with a decreased frequency of mastitis and SCC [61,62,63], likely due to an improved immune system [18,64]. The data from the present experiment and our previous experiment with transition sheep [54] did not confirm those prior observations. In the present experiment, we detected a slight improvement in phagocytosis prepartum, but only in Holstein heifers fed with Se-biofortified hay. The same cows, however, showed a decrease in the proportion of lymphocytes postpartum, which could have contributed to the possible reduction in the production of immunoglobulins, as suggested by the decline in plasma globulins. Those data do not support the previously observed increase in the production of immunoglobulins by Se supplementation [65]. The lack of increase in immunoglobulins by feeding Se-biofortified hay was confirmed by data on immunoglobulin in the colostrum and serum of the calves in the present experiment. Also, in sheep supplemented with Se-yeast, we did not observe an effect on immunoglobulin in the colostrum and serum of the offspring [66]. Similarly, the immunoglobulins in the serum of beef calves were not affected by feeding the dams with the same Se-enriched hay we used in our experiment [67].

Holstein cows receiving Se-biofortified hay had greater phagocytosis of leukocytes at −10 DFC, suggesting a better capacity of leukocytes to cope with pathogens while approaching parturition. Improved leukocyte functions with Se are consistent with the increased chemotactic and respiratory burst of neutrophils documented in sheep supplemented with Se nanoparticles compared to sodium selenite [68]. The numerically lower abundance of WBCs, mainly due to neutrophils in Holstein cows receiving Se-biofortified hay, may suggest, together with the haptoglobin data, a mitigation of inflammation by Se administration in those cows.

### 4.3. Se-Biofortified Hay Had an Unclear Effect on Inflammation and Oxidative Stress

Supplementation of dairy cows with several organic forms of Se during the transition period enhanced the antioxidant response [64]. The cows in our experiment had an increased GPx activity in whole blood [31], indicating a better antioxidative response. However, the analysis of the blood plasma did not support such a conclusion, as none of the antioxidant-related parameters measured was affected by feeding Se-biofortified hay, similar to what we have previously observed with transition sheep supplemented with Se-yeast [54]. Surprisingly, we observed an increase in ROM. Supplementation of Se is usually associated with a decrease in reactive oxygen molecules [69], as many of the selenoproteins reduce reactive oxygen molecules, including GPx and Selenoprotein W [70], both of which were upregulated in the liver during the prepartum stage. The reason for such observation is unclear; however, inflammation and oxidative stress are often associated [71]. Interestingly, in our experiment, the positive acute phase protein ceruloplasmin was one of the most affected and upregulated parameters by feeding Se-biofortified hay. This protein transports Cu and has anti-inflammatory and antioxidant functions [72]. Thus, the increase in ceruloplasmin might indicate an increase in inflammation, but can also indicate an increase in antioxidative response. The increase in inflammation appears to be marked by an early rise in haptoglobin prepartum, but only in Jersey cows, while Holsteins had the opposite response. A possible higher inflammatory response in Jersey vs. Holstein cows fed Se-biofortified hay is supported by higher numerical values of ROM. However, none of the negative acute phase proteins (e.g., albumin, paraoxonase) or other parameters related to inflammation (e.g., myeloperoxidase, Zn) [36] were affected, indicating that if feeding Se-biofortified hay increased inflammation more significantly, this had a minor physiological effect. An increase in the positive acute phase serum amyloid A early postpartum was observed in Holstein dairy cows replete with Se prepartum and supplemented with Se-yeast during late gestation [17]; however, in the same work, a concomitant increase in serum albumin was observed in animals receiving Se-yeast, which was not confirmed in our experiment.

### 4.4. Se-Biofortified Hay Affects Mineral Metabolism and Kidney Function in Cows

As our previous data documented, Sel cows have a greater blood Se concentration and a heightened GPx availability [31]. Lower creatinine levels detected throughout this experiment suggest an improved glomerular filtration rate [73] in the kidneys of cows receiving Se-biofortified hay. We could speculate that the lowered creatinine concentration could depend on the positive effect of GPx on kidney function [74].

The kidney is directly involved in converting vitamin D to its active form [75], while selenoproteins and GPx improve bone formation by heightening osteoblast differentiation and lowering osteoclast activity [76]. A greater osteoclast abundance and more efficient activation of vitamin D by the kidneys could account for the greater concentration of circulating Ca detected in Sel cows, as osteoclasts are primarily involved in replenishing the circulating Ca pool at the onset of lactation [77]. Interestingly, lowered plasma Ca was detected at 14 DFC in Jersey cows receiving Sel-biofortified hay. In the same animals, we detected a higher expression of *SELENOW* in the liver compared to the Ctr, a transcript coding for a protein whose function is not fully clear but that might be involved in bone metabolism [76].

Although the levels of Mg and Ca were in the normal ranges [78], the lower plasma Mg detected in cows receiving Se-biofortified hay throughout the study (especially Holstein cows) could be due to a lower Mg intake or absorption, which has been associated with increased risk for developing hypocalcemia [77], although none of our cows had clinical hypocalcemia. We speculate that Holstein cows could have been penalized for the amount of dietary Mg administered during the trial due to their more significant metabolic requirements [79]. Despite that, the greater efficiency of rumen fermentations of Holstein cows allowed a faster recovery of homeostasis compared to Jersey, as reflected by the higher Ca concentration detected in Holstein cows fed Se-biofortified hay at 14 DFC.

### 4.5. Transcriptomics Data Confirmed the Mild Effect of Feeding Se-Biofortified Hay on the Cow’s Liver

The above observation of a mild effect on the physiology of pregnant dairy heifers by feeding Se-biofortified hay is supported by the modest effect on the transcriptome in the liver of Jersey cows. With the relatively modest impact overall, we found very few DEGs in the postpartum period, with most of the difference in hepatic gene expression concentrated in prepartum samples. This is consistent with the measured selenium concentration in the liver of our Jersey cows, which was higher prepartum than postpartum [31].

The lack of an effect of Se-biofortified hay on the liver’s transcriptome appears to agree with existing literature. A recent study conducted on grazing sheep indicated that 53 DEGs were found in animals fed a selenium-deficient diet compared to animals fed a diet with adequate selenium levels [80]. Interestingly, the most impacted gene in that study was *SELENOW*, which was downregulated in selenium-deficient sheep. The same gene was strongly upregulated in the Sel cows in our study. Along with *SELENOW*, the transcription of *GPX1* was upregulated by feeding Se-biofortified hay to Jersey cows. Both genes are directly tied to selenium metabolism and have been previously shown to respond to selenium supplementation [81]. The connection between the other DEGs and selenium supplementation is less clear. Some of the DEGs may indicate that the cellular stress response (e.g., *ATF5* [82]) and redox-sensitive signaling (e.g., *PKAR2B* [83]) are involved in the liver’s response to selenium; these genes were also found to be upregulated in an in vitro model of the human prostate treated with Se [84].

### 4.6. Se-Biofortified Hay Administered to Pregnant Heifers Has a Minor Effect on Newborn Calves

Feeding pregnant dairy heifers with Se-biofortified hay in the last 40 days of pregnancy did not affect the concentration of immunoglobulins in colostrum, but the higher plasma concentration of Se found in Sel calves [31] indicates that the dietary treatment received by the mother affected the Se status of the offspring, probably due to the combined contribution of placental and colostrum transfer [85]. A heightened absorption of immunoglobulins in newborn calves was expected based on previous data, documenting supplemental Se as an effective treatment to improve the passive immune transfer in calves [22,86]. Surprisingly, this was inconsistent with our findings, as calves born from cows in the Sel group did not have higher concentrations of IgG or IgA in serum, but showed numerically lower concentrations of IgG at 4 DFC. Conversely, the greater relative abundance of total phagocytizing cells found in Holstein calves born from cows fed Se-biofortified hay suggests that the beneficial effects of Se-biofortified hay on the innate immune cells documented in the cows may have been extended to the offspring in our study.

In calves born from cows fed Se-biofortified hay, we detected a more significant concentration of the inflammatory marker haptoglobin, especially at 24 DFC in Holstein calves; however, the liver enzyme GGT was higher in both breeds of calves born from cows fed Se-biofortified hay. The data indicated a possible adverse effect on the liver of the offspring. However, ALP, another liver enzyme, was more favorable in Holstein calves from cows fed Se-biofortified hay. A positive association between circulating liver enzymes and Se intake has been observed in people [87]. In racing horses, an opposite relationship was detected [88]. In sheep, GGT decreased in lambs born from ewes supplemented with organic or inorganic Se [89]. Furthermore, a positive association has been revealed with the level of circulating GGT and the quality of the colostrum and/or passive transfer of immunoglobulins [90,91]. Thus, based on the level of other inflammatory and liver health markers and the association of levels of GGT (and also haptoglobin [91]) with colostrum quality, our data indicate a possible better colostrum in the Sel groups.

It is interesting that several parameters, including paraoxonase, NEFA, bilirubin, and creatinine, tended to differ or were diversely affected by feeding Se-biofortified to the cows based on the breed. Jersey calves showed better outcomes in terms of NEFA, paraoxonase, and bilirubin compared to Holstein calves. Thus, a positive effect of Se on mitigating the mobilization of body fats could be hypothesized for Jersey calves [92]. Although we did not observe any effect on the cows, increased activity of paraoxonase has been reported in humans supplemented with Se [93].

### 4.7. The Metabolism and the Response to Post-Calving Are Different in Holstein and Jersey Heifers

Although the main purpose of the present experiment was not to compare Jersey and Holstein cows, the experimental design allowed for a comparison between the two breeds of peripartum heifers. The two breeds had several differences; some were expected, such as performance-related parameters, but some were not previously explored, such as some of the blood parameters. The higher butterfat content and differences in the FA profile detected in milk from Jersey compared to Holstein cows have been consistently documented in previous studies [94,95], and have been mostly related to genetic traits typical of the two breeds.

The data on NEFA and glucose are indicative of Holstein heifers experiencing a greater energy deficit compared to Jersey heifers when approaching calving, similar to previous observations in which Holstein cows were compared to local breeds [96]. Despite the higher circulating NEFA, the plasma data suggest a better oxidative status in Holstein compared to Jersey heifers, as indicated by the higher FRAP, which reflects the antioxidant power of non-enzymatic compounds (i.e., vitamin C and E, as well as endogenous species such as bilirubin, uric acid, and proteins) [59].

The higher circulating NEFA in Holstein vs. Jersey cows did not negatively affect the liver, as suggested by the higher postpartum glucose levels and an overall better inflammatory and oxidative status [97]. Compared to Jerseys, Holstein heifers had higher phagocytosis of leukocytes, suggesting a greater ability of leukocytes to fight against pathogens, and greater hematocrit, suggesting a greater availability of circulating oxygen [98], possibly improving the respiratory burst activity of leukocytes. Besides this, Holstein heifers had lower plasma ceruloplasmin (a positive acute phase protein [68]) and a less dramatic change in haptoglobin early postpartum, suggesting a lower inflammation and a possible faster resolution of it due to a more efficient immune system, as also suggested by prior data [55]. A more efficient immune system matches with the better health status of the mammary gland of Holstein compared to Jersey heifers, as reflected by lower SCC, while a faster resolution of inflammatory states could partially account for the higher plasma glucose and lower ROM concentration, as activated leukocytes are directly involved in glucose consumption [99] and oxidant species generation [100]. In this context, we could speculate that the greater benefit of Se treatment on immune parameters in Holstein heifers was due to a more efficient immune system compared to Jersey heifers, possibly enlarging the positive effect of supplemental GPx [31] on leukocyte functions. We can speculate that the lower plasma Mg detected in Holstein compared to Jersey heifers throughout the study could have been due to a marginal insufficiency of Mg in the diet to meet their greater metabolic requirements [79].

### 4.8. Holstein and Jersey Calves Are Different

As for the cows, our experiment allowed us to compare the calves between the two breeds. We observed higher BW, greater concentrations of serum immunoglobulins, greater abundance of several plasma analytes related to energy and protein metabolism (i.e., glucose, urea, and creatinine), as well as a greater relative abundance of phagocytizing granulocytes in Holstein vs. Jersey calves, suggesting a higher metabolic status but also a better immune system.

### 4.9. Limitations

Our study presents several limitations. The number of animals used was small, and the two breeds had an unbalanced number of animals between the treatments. Another limitation was the use of pregnant heifers instead of multiparous cows. Thus, it is possible that the beneficial effect of Se supplementation was not detected due to the use of young and healthy animals. Using primiparous instead of multiparous cows could have impacted the trends of plasma biomarkers reflecting inflammation and oxidative stress detected in our study in response to Se treatment. It is well known that primiparous cows are less susceptible to diseases compared to multiparous cows, and it is possible that the beneficial effect of the Se-biofortified hay across the two breeds used was not detected due to the use of healthy animals raised under optimal environmental conditions. The limited number of animals in our OSU Dairy Center and the contemporaneous use of multiparous cows for another experiment prevented the use of a single breed and multiparous cows.

The short-term data collection after parturition (14 days), especially milk components, was also a limitation of the study. This is suggested by the tendency for larger milk yield in Holstein cows when observed for >100 days postpartum.

Several plasma analytes (i.e., NEFA, urea, creatinine, Mg, ALP, and ceruloplasmin) and the relative abundance of total phagocytizing cells were affected by the sex of the calves; however, the calves were not blocked by sex in this experiment.

## 5. Conclusions

The present study revealed that supplementing pregnant heifers with organic Se above the NRC recommendation by using a relatively low amount of Se-biofortified hay—despite improving the levels of Se in whole blood—did not influence the performance or the quality of milk, including the FA profile of butterfat. The treatment also had little effect on metabolic and inflammatory parameters and did not affect the offspring. Furthermore, despite higher GPx activity in blood and plasma, no other parameters related to antioxidant response were affected in dams or calves. However, the data allowed us to infer that the supplementation of pregnant heifers with Se-biofortified hay may be more effective in Holstein heifers, as we observed some improvements in milk yield and the immune status of those animals. As indicated above, a lack of more substantial effects might be due to using primiparous instead of multiparous cows and the low number of animals. It appears sensible to further test the impact of Se-biofortified hay in pluriparous cows.

## Figures and Tables

**Table 1:** Performance of Jersey (n = 5/group) and Holstein (n = 4/group) pregnant dairy heifers supplemented with 1% BW of Se-biofortified alfalfa hay (Sel) or no biofortified alfalfa hay (Ctr) from −40 through 14 days from calving.

Parameter	Holstein		Jersey		*SEM ^1^*	*p*-Value ^1^					
	Ctr	Sel	Ctr	Sel		*Se*	*Br*	*Se × Br*	*Se × T*	*Br × T*	*Se × Br × T*
BW kg	559	555	479	473	*23.5*	0.83	<0.01	0.98	0.99	0.06	0.99
BCS	2.81	2.78	2.71	2.86	*0.07*	0.34	0.62	0.37	0.64	<0.01	0.41
DMI kg/d ^2^	12.1 ^a^	12.7 ^a^	10.4 ^b^	10.5 ^b^	*0.29*	0.18	<0.01	<0.01	0.82	<0.01	0.16
DMI/BW % ^2^	2.22	2.23	2.14	2.31	*0.06*	0.08	0.55	0.44	0.90	<0.01	0.24
Milk Yield Kg/d ^3^	20.4 ^b^	22.7 ^a^	19.6 ^bc^	17.9 ^c^	*0.70*	0.70	<0.01	<0.01	<0.01	<0.01	0.02
Milk Yield Kg/d ^2,4^	27.2	29.1	24.7	24.7	*0.68*	0.15	<0.01	0.15	0.02	0.20	<0.01
ECM kg/d ^5^	19.2	20.9	21.0	17.8	*1.68*	0.64	0.68	0.15	0.21	0.37	0.73
*Milk composition*											
Fat %	2.83	2.82	4.08	3.45	*0.47*	0.48	0.05	0.50	0.99	0.08	0.48
Protein %	3.59	3.73	3.74	3.82	*0.11*	0.34	0.29	0.77	0.22	0.62	0.69
Lactose %	4.62	4.65	4.53	4.47	*0.08*	0.85	0.09	0.56	0.48	0.59	0.70
SNF %	9.34	9.44	9.50	9.33	*0.12*	0.76	0.81	0.27	0.23	0.53	0.15
SCC (log_2_)	7.17	5.07	8.28	7.71	*0.83*	0.11	0.03	0.34	0.56	0.47	0.21
MUN mg/dL	13.2	13.6	15.1	16.4	*1.01*	0.42	0.02	0.66	0.83	0.33	0.25
Fat g/d	593	619	719	632	*99.1*	0.75	0.47	0.56	0.64	0.01	0.51
Protein g/d	750	843	735	656	*45.8*	0.87	0.03	0.07	0.23	0.72	0.13
Lactose g/d	967	1070	878	779	*55.1*	0.97	<0.01	0.07	0.02	0.14	0.35
Conductivity mS	9.40	9.24	9.27	9.31	*0.13*	0.64	0.81	0.41	0.02	0.14	0.04
Activity min/d	108	114	152	151	*14.1*	0.86	<0.01	0.81	0.84	0.13	0.69

^1^ Se = Se-biofortified alfalfa treatment; Br = Breed; T = Time with the largest SEM reported; ^2^ The data were analyzed as weekly average; ^3^ Up to14 days postpartum with daily milk yield; ^4^ Up to 110 days postpartum with daily milk yield averaged weekly; ^5^ Energy-Corrected Milk = kg milk * (38.3 × kg fat + 24.2 × kg protein + 15.71 × kg lactose + 20.7)/3140 [45]. Different letters denote significant (*p* < 0.05) effect between groups.

**Table 2:** Plasma parameters in Jersey (n = 5/group) and Holstein (n = 4/group) pregnant dairy heifers supplemented with 1% BW of Se-biofortified alfalfa hay (Sel) or no biofortified alfalfa hay (Ctr) from −40 through 14 days from calving.

Parameter	Holstein		Jersey		*SEM ^1^*	*p*-Value ^1^					
	Ctr	Sel	Ctr	Sel		*Se*	*Br*	*Se × T*	*Se × Br*	*Br × T*	*Se × Br × T*
Hematocrit *V*/*V*%	33.8	34.1	32.7	32.2	*0.52*	0.85	<0.001	0.56	0.38	0.54	0.64
*Metabolism*											
NEFA mmol/L	0.55	0.59	0.48	0.40	*0.07*	0.72	0.07	0.79	0.37	0.02	0.63
NEFA/Albumin	1.12	1.13	0.94	0.70	*0.13*	0.35	0.03	0.56	0.33	0.07	0.25
BHB mmol/L	0.58	0.66	0.67	0.67	*0.07*	0.55	0.45	0.49	0.55	0.28	0.66
Glucose mmol/L	4.36	4.40	4.34	4.26	*0.09*	0.80	0.41	0.55	0.47	0.01	0.96
Cholesterol mmol/L	2.16	2.27	2.27	2.28	*0.13*	0.59	0.15	0.92	0.67	0.28	0.99
Urea mmol/L	5.0	5.5	5.2	4.9	*0.28*	0.67	0.28	0.64	0.14	0.28	0.86
*Inflammatory status and immune system*											
Ceruloplasmin µmol/L	2.55	2.92	2.81	2.98	*0.10*	0.01	<0.01	0.28	0.32	0.36	0.09
Haptoglobin g/L	0.55	0.46	0.68	0.68	*0.10*	0.59	0.14	0.29	0.60	0.63	0.01
Myeloperoxidase U/L	380	412	382	411	*24.6*	0.22	0.82	0.39	0.93	0.89	0.34
Total protein g/L	73.7 ^a^	70.6 ^b^	70.6 ^b^	73.2 ^a^	*0.96*	0.79	0.63	0.68	0.00	0.54	0.66
Albumin g/L	35.6	35.8	35.9	36.7	*0.72*	0.46	0.33	0.17	0.66	0.59	0.10
Globulin g/L	38.3 ^a^	34.5 ^b^	34.8 ^b^	36.8 ^ab^	*1.00*	0.33	0.33	0.77	0.01	0.56	0.83
Paraoxonase U/mL	71.9	77.2	78.0	79.5	*4.00*	0.38	0.76	0.80	0.61	0.34	0.99
Albumin/Globulin	0.95	1.06	1.05	1.00	*0.04*	0.44	0.21	0.68	0.08	0.43	0.62
*Liver status*											
AST/GOT U/L	97.0	95.6	97.6	96.5	*3.18*	0.68	0.77	0.69	0.95	0.02	0.57
GGT U/L	20.8	21.0	22.3	22.3	*1.57*	0.94	0.79	0.54	0.94	0.15	0.98
ALP U/L	70.9	88.4	74.4	81.4	*6.73*	0.09	0.43	0.30	0.42	0.85	0.03
Bilirubin µmol/L	4.29	4.50	3.47	3.61	*0.44*	0.68	0.06	0.75	0.93	0.25	0.78
*Oxidative status*											
Thiol Groups µmol/L	339	341	348	343	*10.7*	0.89	0.08	0.52	0.69	0.12	0.64
ROMt mg H_2_O_2_/100 mL	14.0	14.8	14.3	16.2	*0.61*	0.02	<0.01	0.55	0.33	0.57	0.28
FRAP µmol/L	145.4	147.4	147.4	141.1	*6.38*	0.73	<0.01	0.68	0.50	0.14	0.82
AOPP µmol/L	33.5	33.2	32.8	31.5	*1.37*	0.52	0.03	0.50	0.69	0.57	0.86
ROM/FRAP	0.105	0.096	0.105	0.121	*0.01*	0.61	0.01	0.46	0.09	0.01	0.33
*Minerals*											
Ca mmol/L	2.45	2.48	2.43	2.48	*0.03*	0.11	0.58	0.22	0.67	0.89	0.02
Free Ca mmol/L	1.21	1.22	1.20	1.22	*0.01*	0.09	0.26	0.29	0.61	0.60	0.10
Mg mmol/L	0.99	0.95	1.09	1.05	*0.03*	0.17	<0.01	0.28	0.97	0.54	0.01
*Kidney function*											
Creatinine µmol/L	96.4	92.9	88.9	85.6	*1.52*	0.01	<0.01	0.44	0.93	0.16	0.43
Urea/Creatinine	49.4	57.3	60.5	61.6	*2.90*	0.08	0.04	0.67	0.19	0.61	0.89

^1^ Se = Se-biofortified alfalfa treatment; Br = Breed; T = Time with the largest SEM reported. Different letters denote significant (*p* < 0.05) effect between groups.

**Table 3:** Whole blood count and phagocytizing capacity of circulating leukocytes in Holstein and Jersey dairy cows receiving 1 kg/100 kg BW of Se-biofortified alfalfa hay containing 3.25 mg Se/kg DM (Sel), or of a standard alfalfa hay containing 0.43 mg Se/kg DM (Ctr) from −40 through 14 days from calving.

Parameter	Holstein		Jersey		*SEM ^1^*	*p*-Value ^1^					
	Ctr	Sel	Ctr	Sel		*Se*	*Br*	*Se × T*	*Se × Br*	*Br × T*	*Se × Br × T*
WBC 10^3^/mL	13.6	9.99	10.8	10.2	1.26	0.06	0.24	0.31	0.17	0.04	0.11
Lymphocytes 10^3^/mL ^2^	8.41	5.78	6.28	5.80	1.19	0.15	0.32	0.27	0.31	0.27	0.60
Neutro+mono 10^3^/mL ^2^	5.02	4.16	4.27	4.00	0.54	0.23	0.33	0.82	0.53	0.11	0.12
Lymphocytes % ^3^	61.7	55.7	57.4	56.4	5.77	0.50	0.72	0.35	0.62	0.45	0.84
Neutrophils % ^3^	36.9	43.6	40.5	40.1	5.32	0.50	0.99	0.21	0.45	0.52	0.91
Monocytes % ^3^	1.38	0.90	2.16	3.50	1.52	0.75	0.22	0.37	0.50	0.16	0.83
*Phagocytosis %*											
Leukocytes ^3^	26.4	37.2	20.3	21.5	4.65	0.188	0.03	0.52	0.29	0.43	0.05
Neutrophils ^3^	52.8	72.1	40.7	43.7	9.07	0.215	0.03	0.33	0.36	0.96	0.19

^1^ Se = Se-biofortified alfalfa treatment; Br = Breed; T = Time with the largest SEM reported; ^2^ Estimated using the % for each population obtained using flow cytometer. ^3^ Obtained using antibodies (CH138A for granulocytes and CAM36A for monocytes). The lymphocytes were obtained by subtracting the former two from all nucleated cells detected.

**Table 4:** Plasma parameters in calves born from Jersey (n = 5/group) and Holstein (n = 4/group) pregnant dairy heifers supplemented with 1% BW of Se-biofortified alfalfa hay (Sel) or no biofortified alfalfa hay (Ctr) for 40 days prior to calving.

Parameter	Holstein		Jersey		*SEM ^1^*		*p*-Value ^1^					
	Ctr	Sel	Ctr	Sel		*T ^2^*	*Se*	*Br*	*Se × T*	*Se × Br*	*Br × T*	*Se × Br × T*
*Metabolism*												
NEFA mmol/L	0.15	0.28	0.25	0.15	0.06	↓	0.84	0.86	0.43	0.09	0.73	0.26
NEFA/Albumin	0.30	0.63	0.60	0.34	0.14	↓	0.83	0.98	0.46	0.09	0.52	0.30
BHB mmol/L	0.090	0.091	0.127	0.087	0.02	↔	0.36	0.45	0.68	0.39	0.16	0.39
Glucose mmol/L	9.22	8.95	7.05	8.01	0.42	↓	0.44	0.01	0.11	0.22	0.06	0.06
Cholesterol mmol/L	2.66	2.40	2.13	2.33	0.32	↑	0.93	0.41	0.79	0.54	0.20	0.743
Urea mmol/L	3.89	4.35	3.56	2.72	0.30		0.56	0.01	0.57	0.09	0.12	0.33
Ceruloplasmin µmol/L	1.56	1.86	2.05	1.92	0.20	↑	0.69	0.24	0.50	0.38	0.38	0.06
Haptoglobin g/L	0.41	0.60	0.38	0.42	0.04	↑	0.01	0.03	0.11	0.14	0.01	0.15
Myeloperoxidase U/L	385	399	260	356	37.9	↔	0.19	0.07	0.59	0.37	0.35	0.84
Total protein g/L	70.7	73.1	67.3	71.3	2.33	↓	0.20	0.33	0.71	0.77	0.99	0.44
Albumin g/L	31.5	30.9	30.2	30.4	0.40	↑	0.65	0.07	0.53	0.36	0.06	0.13
Globulin g/L	39.1	42.1	37.1	40.8	2.33	↓	0.19	0.54	0.61	0.88	0.62	0.26
Paraoxonase U/mL	42.5 ^a^	26.7 ^b^	24.1 ^b^	33.2 ^ab^	3.29	↑	0.34	0.13	0.21	0.01	0.87	0.02
Albumin/Globulin	0.84	0.75	0.87	0.79	0.05	↑	0.14	0.58	0.67	0.89	0.32	0.45
*Liver status*												
AST/GOT U/L	55.7	53.7	57.5	53.6	6.50	↓	0.67	0.91	0.62	0.90	0.90	0.35
GGT U/L	351	1530	820	1335	285	↓	0.01	0.67	0.50	0.33	0.25	0.93
ALP U/L	344	385	405	352	58.4	↓	0.92	0.84	0.08	0.49	0.02	0.04
Bilirubin µmol/L	3.04	10.63	6.84	2.81	2.41	↓	0.49	0.46	0.63	0.06	0.99	0.33
*Oxidative status*												
Thiol Groups µmol/L	338	330	295	304	20.5	↔	0.97	0.15	0.48	0.71	0.47	0.08
ROMt mg H_2_O_2_/100 mL	10.9	13.4	13.3	12.4	1.24	↑	0.56	0.62	0.59	0.26	0.73	0.19
FRAP µmol/L	125	140	138	122	13.1	↓	0.97	0.87	0.29	0.30	0.92	0.19
AOPP µmol/L	54.8	55.7	63.1	67.0	6.82	↔	0.74	0.21	0.27	0.85	0.15	0.09
ROM/FRAP	0.090	0.114	0.115	0.104	0.01	↑	0.68	0.64	0.46	0.33	0.99	0.07
*Minerals*												
Ca mmol/L	3.09	3.05	3.03	3.02	0.06	↓	0.71	0.55	0.63	0.86	0.86	0.57
Free Ca mmol/L	1.56	1.55	1.55	1.54	0.03	↓	0.77	0.67	0.75	0.98	0.93	0.77
Mg mmol/L	0.86	0.97	0.95	0.90	0.05	↓	0.49	0.93	0.32	0.14	0.71	0.25
*Kidney function*												
Creatinine µmol/L	86.4	93.5	79.1	69.1	3.90	↓	0.72	0.00	0.47	0.08	0.71	0.35
Urea/Creatinine	45.6	45.8	46.3	41.6	2.78	↑	0.46	0.57	0.50	0.47	0.09	0.42

^1^ Se = Se-biofortified alfalfa treatment; Br = Breed; T = Time; with the largest SEM reported. ^2^ Change from 2 to 24 d (↓ decrease; ↑ increase), if significant (*p* < 0.05). ↔ when change is not significant. Different letters denote significant (*p* < 0.05) effect between groups.

**Table 5:** Leukocyte differential and phagocytosis capacity in calves born from Holstein and Jersey dairy cows receiving 1 kg/100 kg BW of Se-biofortified alfalfa hay containing 3.25 mg Se/kg DM (Sel) or standard alfalfa hay containing 0.43 mg Se/kg DM (Ctr) for the last 40 days of pregnancy. The concentration of immunoglobulins in the colostrum and serum of calves is also shown.

Parameter	Holstein		Jersey		*SEM ^1^*	*p*-Value ^1^					
	Ctr	Sel	Ctr	Sel		*Se*	*Br*	*Se × T*	*Se × Br*	*Br × T*	*Se × Br × T*
WBC 10^3^/mL	10.1	10.7	10.9	8.32	1.45	0.44	0.60	0.53	0.27	0.07	0.84
Lymphocytes 10^3^/mL ^2^	5.59	4.96	6.26	4.73	0.62	0.06	0.71	0.06	0.47	0.03	0.76
Neutro + mono 10^3^/mL ^2^	4.51	5.63	4.29	3.70	0.93	0.75	0.25	0.97	0.37	0.33	0.86
Lymphocytes % ^3^	64.8	59.1	61.5	63.9	*4.32*	0.68	0.85	0.04	0.31	0.07	0.21
Neutrophils % ^3^	32.6	35.7	35.1	33.0	*4.20*	0.90	0.99	0.05	0.49	0.05	0.40
Monocytes % ^3^	2.87	4.85	3.40	3.14	*1.58*	0.57	0.69	0.05	0.46	0.04	<0.01
*Phagocytosis %*											
Leukocytes ^3^	24.5	33.3	18.4	20.8	5.68	0.28	0.12	0.01	0.57	0.46	0.02
Neutrophils ^3^	61.7	77.5	37.5	37.5	12.3	0.48	0.02	0.03	0.51	0.39	0.46
*Immunoglobulins*											
Colostrum											
IgG mg/mL	0.58	0.57	0.38	0.37	0.10	0.96	0.06		0.99		
IgA mg/mL	4.64	5.45	4.21	3.92	0.69	0.70	0.16		0.42		
Calves’ serum											
IgG mg/mL	0.22	0.40	0.17	0.19	0.08	0.42	<0.01	0.07	0.22	0.43	0.02
IgA mg/mL	3.94	3.08	2.10	2.32	0.44	0.15	0.07	0.29	0.27	0.21	0.64

^1^ Se = Se-biofortified alfalfa treatment; Br = Breed; T = Time; with the largest SEM reported. ^2^ Estimated using the % for each population obtained using a flow cytometer. ^3^ Obtained using antibodies (CH138A for granulocytes and CAM36A for monocytes). The lymphocytes were obtained by subtracting the former two from all nucleated cells detected.

## Data Availability

Data are available from the corresponding author upon reasonable request.

## Supplementary Materials

The following supporting information can be downloaded at https://www.mdpi.com/article/10.3390/ani15131866/s1. Figure S1. Experimental design and sampling. The Selenium group received 1% BW of Se-biofortified alfalfa (3.25 ppm of Se) from −40 to 14 days relative to parturition, while the Control group received alfalfa without biofortification (0.43 ppm of Se). Figure S2. Feed intake in Holstein and Jersey cows during the trial. Figure S3. Blood parameters affected by the Breed×Time interaction. Figure S4. Immune cell parameters that were affected by various interactions. Figure S5. Immune cells in calves born from Holstein and Jersey dairy cows receiving 1 kg/100 kg BW of a Se biofortified alfalfa hay containing 3.25 mg Se/kg DM (Sel), or of a standard alfalfa hay containing 0.43 mg Se/kg DM (Ctr) from −40 through 14 days from calving. Figure S6. Abundance of circulating immunoglobulin G and A in calves born from Holstein and Jersey dairy cows receiving 1 kg/100 kg BW of a Se biofortified alfalfa hay containing 3.25 mg Se/kg DM (Sel), or of a standard alfalfa hay containing 0.43 mg Se/kg DM (Ctr) from -40 through 14 days from calving. Table S1. Chemical characteristics of alfalfa hays used for the present experiment. Table S2. Composition and chemical characteristics of the total mixed rations used in the present experiment. Table S3. Fatty acid profile (mg/100 mg) in milk fat of Jersey and Holstein primiparous cows supplemented with 1% BW of Se-biofortified alfalfa hay (Selenium) or no biofortified alfalfa hay (Control) from 40 days prior expected parturition to 14 days after calving. Data are the average of three milk samples (3, 7, and 14 DIM).

## Abbreviations

The following abbreviations are used in this manuscript:

ALP	alkaline phosphatase
AOPP	advanced oxidation protein products
BCS	body condition score
BHB	beta-hydroxybutyric acid
DIM	day in milk
DMI	dry matter intake
GPx	glutathione peroxidase
MPO	myeloperoxidase
MUN	milk urea nitrogen
NEFA	non-esterified fatty acids
PUFA	polyunsaturated fatty acids
ROS	reactive oxygen species
SCC	somatic cell count
SNF	solid non-fat
TMR	total mixed ration
