# Supplementary material for: Selenium-Biofortified Alfalfa Hay Supplemented to Jersey and Holstein Dairy Heifers During the Peripartum Period: Effects on Dams and Their Offspring
Source: Animals (Basel). 2025 Jun 24;15(13):1866. doi: 10.3390/ani15131866 (PMC12248524; doi:10.3390/ani15131866)
Supplement: Supplementary file 1 [file animals-15-01866-s001.zip › Suppl. File S1.pdf]

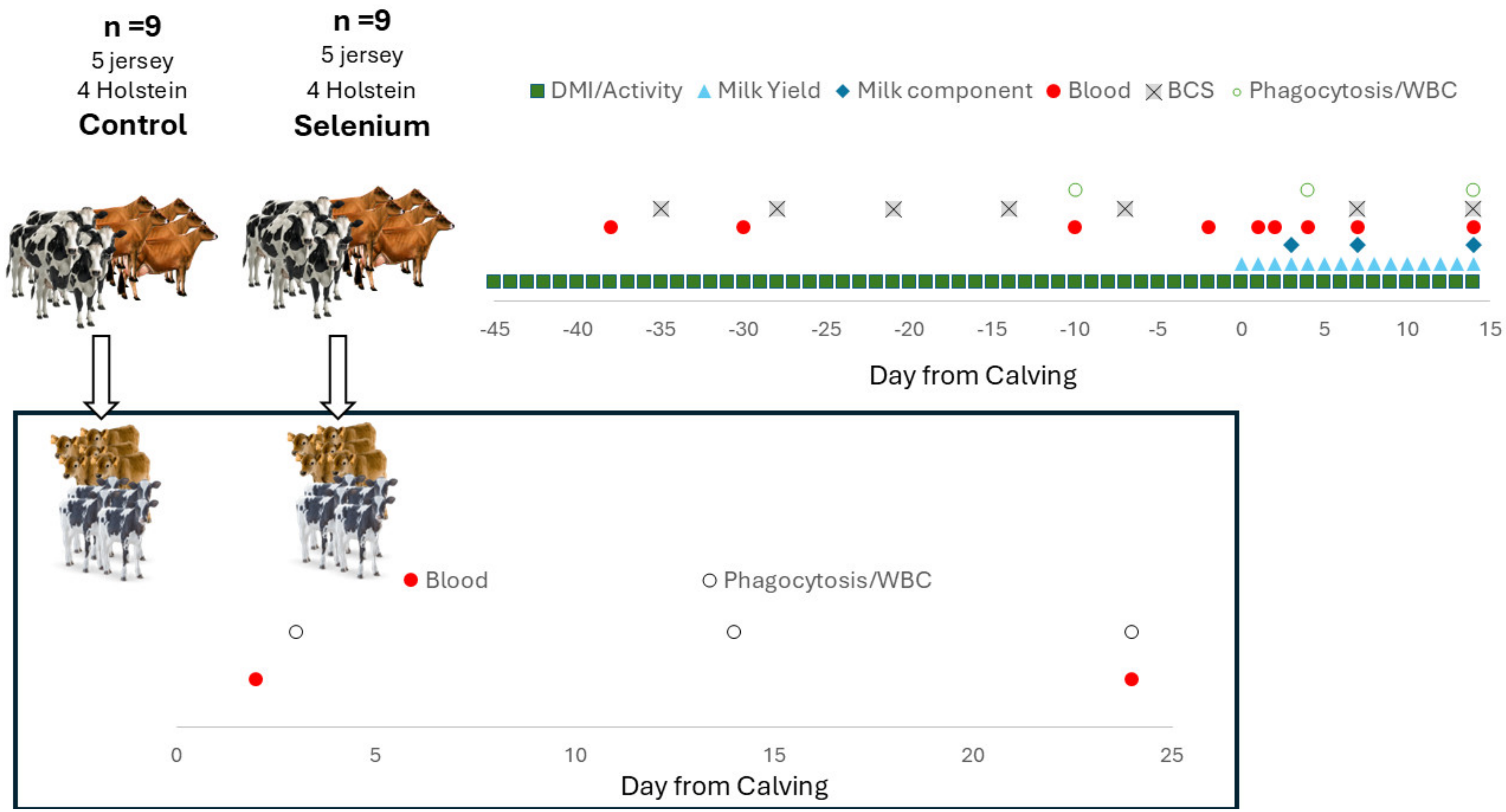

Figure S1. Experimental design and sampling. The Selenium group received 1% BW of Se-biofortified alfalfa (3.25 ppm of Se) from -40 to 14 days relative to parturition, while the Control group received alfalfa without biofortification (0.43 ppm of Se).

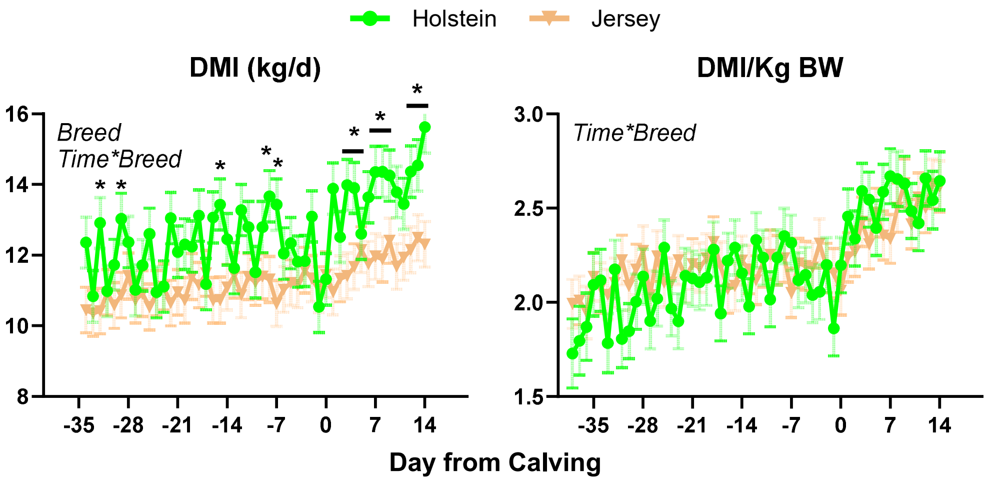

Figure S2. Feed intake in Holstein and Jersey cows during the trial. Significant (P<≤0.05) differences between breeds at the same time point are marked with *.

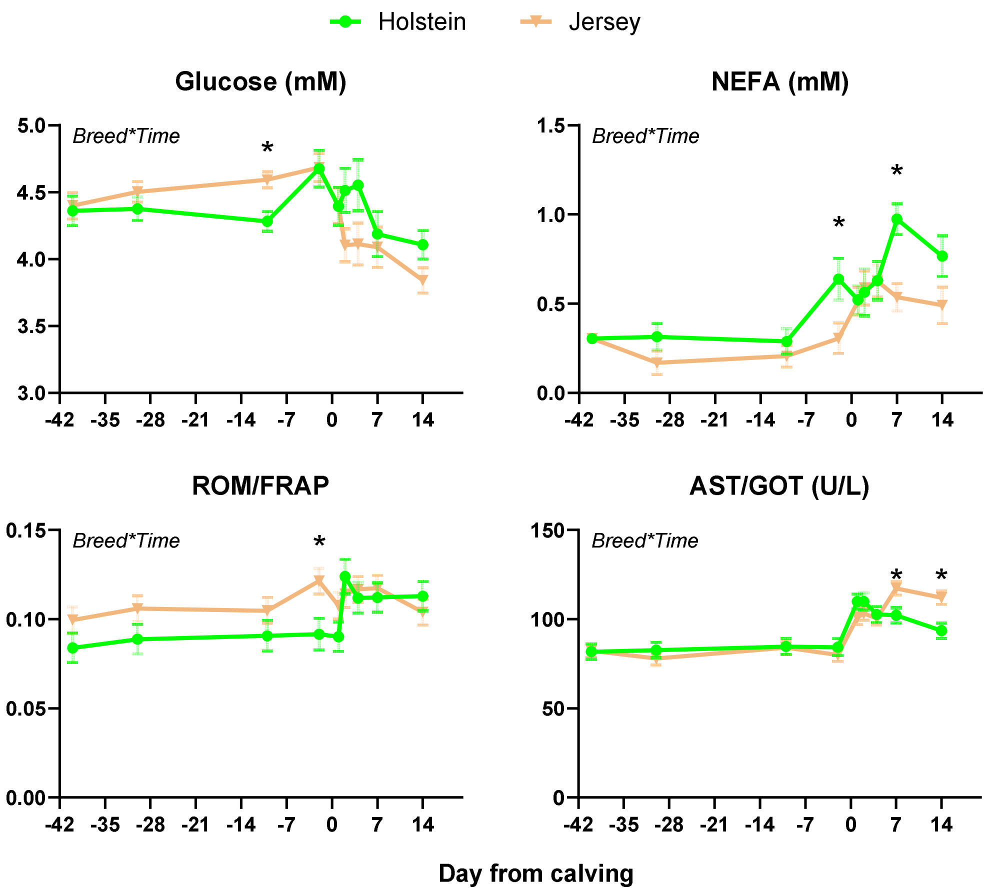

Figure S3. Blood parameters affected by the Breed×Time interaction. Significant (P<≤0.05) differences between breeds at the same time point are marked with *.

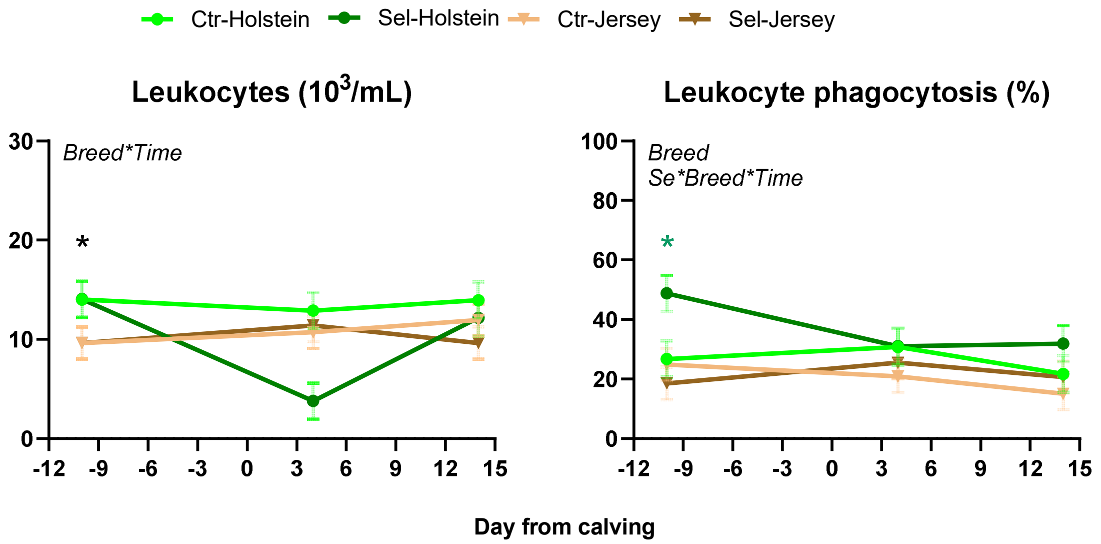

Figure S4. Immune cell parameters that were affected by various interactions. Significant (P<≤0.05) differences between breeds at the same point are marked with *. When a full Se*Breed*Time interaction, the * (green) indicates the difference between Se-biofortified treated and control Holstein cows.

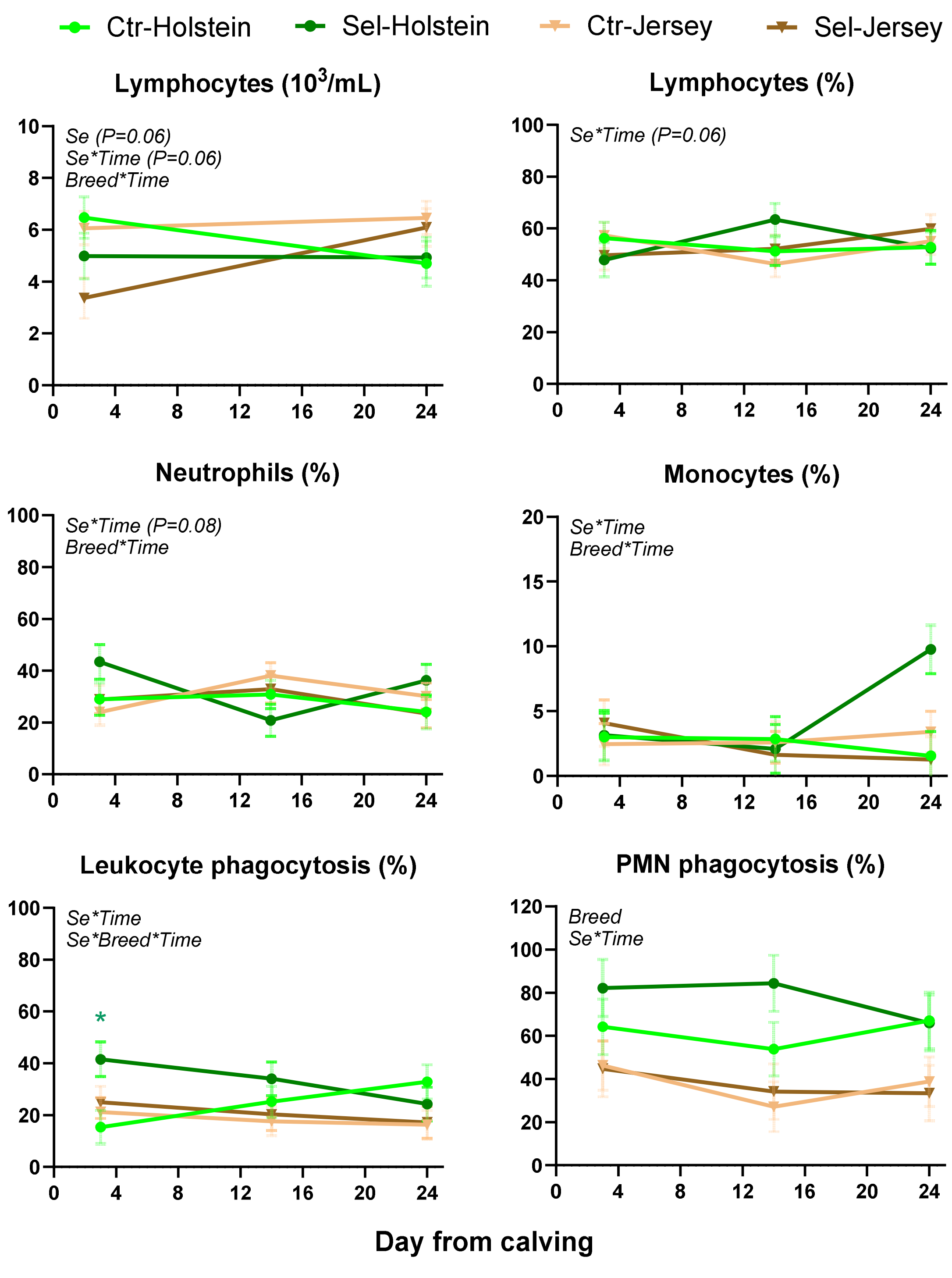

Figure S5. Immune cells in calves born from Holstein and Jersey dairy cows receiving 1 kg/100 kg BW of a Se biofortified alfalfa hay containing 3.25 mg Se/kg DM (Sel), or of a standard alfalfa hay containing 0.43 mg Se/kg DM (Ctr) from -40 through 14 days from calving. # indicates a tendency for the Jersey groups. * (green) indicates the difference between Se-biofortified treated and control Holstein cows.

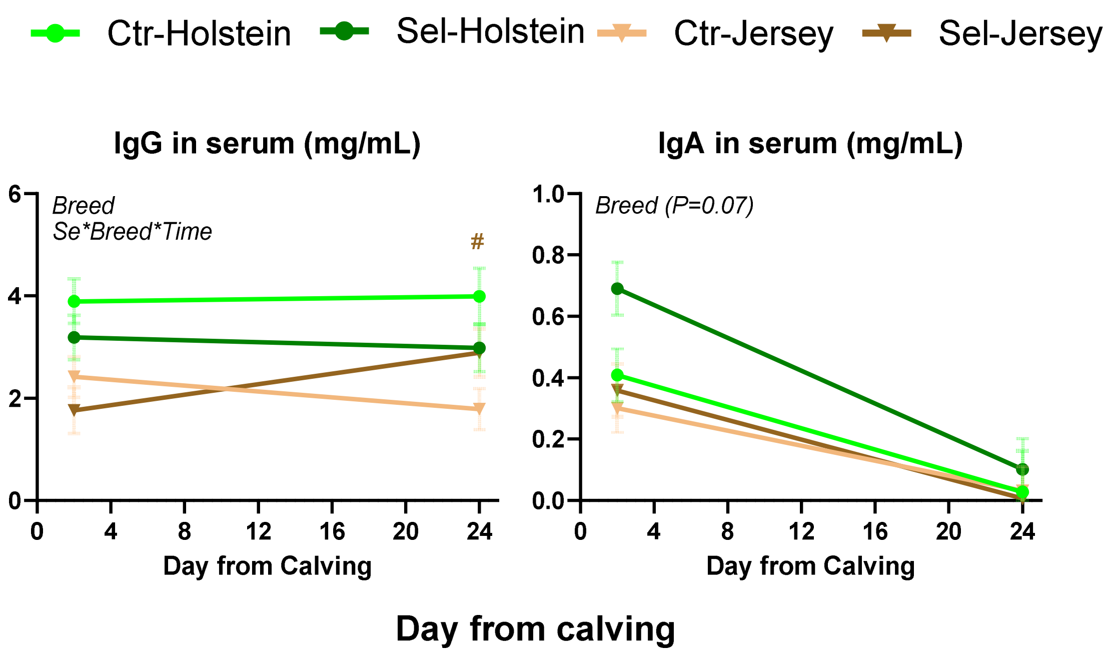

Figure S6. Abundance of circulating immunoglobulin G and A in calves born from Holstein and Jersey dairy cows receiving 1 kg/100 kg BW of a Se biofortified alfalfa hay containing 3.25 mg Se/kg DM (Sel), or of a standard alfalfa hay containing 0.43 mg Se/kg DM (Ctr) from -40 through 14 days from calving. # indicates a tendency for the Jersey groups.

Table S1. Chemical characteristics of alfalfa hays used for the present experiment

	**Control Hay**	**Medium Se hay**	**High Se hay**
DM, %	88.6	89.6	88.9
Nutrient, DM basis			
CP, %	21.8	19.8	20.8
ADF, %	31.6	35.9	31.9
NDF, %	37.9	44.2	40.4
NFC, %	28.3	24.0	26.8
NEL, Mcal/kg	0.63	0.60	0.62
Relative Feed Value	158	128	148
Minerals, DM basis			
Ca, %	1.52	1.35	1.42
P, %	0.22	0.22	0.24
Mg, %	0.38	0.37	0.24
K, %	1.96	1.97	1.95
Na, %	0.092	0.094	0.097
S, %	0.34	0.31	0.33
Fe, ppm	258	723	305
Zn, ppm	17	16	17
Cu, ppm	11	10	10
Mn, ppm	42	51	46
Mo, ppm	< 0.1	< 0.1	< 0.1
Se, ppm	0.43	2.2	4.4

Table S2. Composition and chemical characteristics of the total mixed rations used in the present experiment

Item	Dry cows	Lactating cows
Ingredient, %		
Alfalfa	-	13.6
Corn Silage	40	39.6
Grass Silage	45	26.1
Grass Hay	15	-
Corn/Barley	-	14.4
Soybean Meal	-	5.6
Mineral Supplement[1]	-	0.7
Dry Matter (DM)[2]	41.40± 3.68	50.00±3.96
NE_L; Mcal/kg of DM	1.54±0.00	1.67±0.01
Chemical composition, % DM		
Crude protein	9.00±0.14	18.3±0.99
NDF	52.3±0.8	32.5±4.0
ADF	30.1±0.3	19.6±3.3
Calcium	0.33±0.02	0.59±0.09
Phosphorus	0.24±0.01	0.42±0.08
Magnesium	0.19±0.01	0.25±0.03
Potassium	1.44±0.47	1.87±0.50
Sodium	0.08±0.07	0.06±0.01
Sulfur	0.14±0.01	0.22±0.01
Iron (ppm)	1021±238	536±133
Zinc (ppm)	28.5±0.7	44.0±21.2
Copper (ppm)	6.00±1.41	11.50±4.9
Manganese (ppm)	140.5±33.2	58.5±7.8
Molybdenum (ppm)	1.00±0.00	1.15±0.21
Selenium (ppm)	0.26±0.17	0.99±0.37

[1] Wilbur-Ellis Feed, LLC, OR (Cat# 1187036). It contains (as %DM) 17.60-21.00 % Ca, 7% P, 8% Mg, 1.65% S, 20-24 ppm Se, 440,000 IU/Kg DM Vitamin A. In addition to the mineral provided via TMR cows also received an individual mineral block without Selenium (Cat#90013, Stockman Trace Mineralized & Salt Brick). The indicated content included a minimum guarantee of 98% NaCl, 4000 ppm Zn, 1600 ppm Fe, 1200 ppm Mn, 260 ppm Cu, 100 ppm I, and 40 ppm Co.

[2]The data are mean±SD of monthly TMR samples collected during the trial

Table S3. Fatty acid profile (mg/100 mg) in milk fat of Jersey and Holstein primiparous cows supplemented with 1% BW of Se-biofortified alfalfa hay (Selenium) or no biofortified alfalfa hay (Control) from 40 days prior expected parturition to 14 days after calving. Data are the average of three milk samples (3, 7, and 14 DIM)

**Fatty acid**	**Holstein**		**Jersey**			**P-value**[1]				
mg/100 mg	**CTR**	**Se**	**CTR**	**Se**	***SEM***	***Se***	***Br***	***Se×T***	***Se×Br***	***Se×Br×T***
C4:0	2.96	3.11	2.88	2.96	*0.23*	0.62	0.62	0.96	0.90	0.51
C6:0	2.43	2.27	2.32	2.15	*0.23*	0.48	0.62	0.73	0.98	0.79
C8:0	1.33	1.19	1.33	1.10	*0.16*	0.26	0.79	0.71	0.79	0.57
C10:0	2.45	2.09	2.35	1.79	*0.33*	0.18	0.57	0.61	0.76	0.61
C11:0	0.16	0.14	0.12	0.11	*0.02*	0.37	0.10	0.90	0.86	0.27
C12:0	2.62	2.22	2.54	1.94	*0.35*	0.17	0.62	0.48	0.77	0.67
C14:0	9.02	8.43	8.46	7.37	*0.84*	0.33	0.35	0.32	0.77	0.80
C14:0hydroxy	0.03	0.02	0.02	0.03	*0.01*	0.86	0.64	0.55	0.50	0.75
C14:1cis7	0.23	0.25	0.20	0.22	*0.02*	0.27	0.10	0.50	0.80	0.74
C14:1cis9	0.49	0.49	0.38	0.36	*0.04*	0.82	0.01	0.79	0.74	0.92
C15:0	1.12	1.13	0.97	0.90	*0.07*	0.70	0.01	0.81	0.58	0.90
C16:0	25.9	25.5	26.2	25.2	*0.77*	0.37	0.97	0.15	0.71	0.60
C16:0anteiso	0.33	0.35	0.31	0.33	*0.02*	0.28	0.24	0.84	0.89	0.50
C16:1trans	0.50	0.51	0.44	0.51	*0.02*	0.10	0.17	0.07	0.23	0.81
C16:1cis9	1.85	1.84	2.11	2.37	*0.19*	0.53	0.05	0.49	0.48	0.58
C16:1cis11	0.05	0.07	0.04	0.06	*0.02*	0.33	0.47	0.19	0.84	0.79
C17:0 anteiso	0.29	0.29	0.14	0.18	*0.04*	0.46	<.01	0.17	0.60	0.10
C17:0	1.08	1.13	1.01	1.07	*0.05*	0.28	0.23	0.30	1.00	0.45
C17:1cis10	0.03	0.04	0.03	0.05	*0.01*	0.31	0.80	0.49	0.62	0.66
C17:1cis12	0.46	0.44	0.48	0.54	*0.05*	0.70	0.33	0.57	0.50	0.78

[1]TRT = Treatment; Br = Breed; T = Time

Table S3. Cont

Fatty acid	Holstein		Jersey		SEM	P-value				
mg/100 mg	CTR	Se	CTR	Se	SEM	Se	Br	Se×T	Se×Br	Se×Br×T
C18:0	12.6	14.2	12.6	13.3	*0.60*	0.08	0.49	0.22	0.53	0.54
C18:0anteiso	0.11	0.12	0.12	0.13	*0.02*	0.75	0.74	0.10	0.89	0.20
C18:1t12	0.37	0.29	0.36	0.37	*0.04*	0.40	0.31	0.08	0.21	0.28
C18:1t13+t14	1.41	1.78	1.32	1.45	*0.07*	<.01	0.01	0.17	0.08	0.47
C18:1cis9	23.1	22.7	24.2	26.4	*2.13*	0.70	0.27	0.49	0.56	0.96
C18:1cis10	0.55	0.58	0.48	0.48	*0.03*	0.60	<.01	0.65	0.43	0.36
C18:1cis11	0.80	0.81	0.98	1.13	*0.08*	0.37	0.01	0.38	0.40	0.64
C18:1cis12	0.21	0.21	0.03	0.00	*0.02*	0.50	<.01	0.32	0.32	0.50
C18:1t16+c14	0.31	0.33	0.47	0.37	*0.04*	0.29	0.02	0.86	0.18	0.66
C18:1cisomer	0.17	0.18	0.21	0.20	*0.04*	0.98	0.48	0.78	0.80	0.56
C18:2	2.00	2.11	1.98	2.00	*0.09*	0.45	0.49	0.87	0.60	0.84
C18:2c9t12	0.06	0.09	0.08	0.10	*0.02*	0.38	0.50	0.99	0.81	0.43
C18:2t9c12	0.19	0.20	0.15	0.11	*0.03*	0.55	0.06	0.66	0.41	0.41
C18:3t9c12c15	0.05	0.03	0.07	0.07	*0.02*	0.58	0.08	0.48	0.41	0.38
C18:3c9t12t15	0.18	0.17	0.16	0.16	*0.01*	0.87	0.17	0.94	0.68	0.71
C18:3n3	0.62	0.68	0.57	0.62	*0.03*	0.09	0.08	0.58	0.92	0.86
C18:3CLN	0.33	0.28	0.31	0.39	*0.02*	0.53	0.06	0.13	0.02	0.04
C19:0	0.08	0.05	0.07	0.10	*0.04*	0.94	0.67	0.92	0.47	0.72
C19:1	0.11	0.10	0.14	0.14	*0.02*	0.93	0.11	0.61	0.91	0.82
C20:0	0.10	0.05	0.06	0.07	*0.02*	0.28	0.30	0.71	0.07	0.62
C20:3t	0.06	0.01	0.07	0.06	*0.02*	0.06	0.11	0.29	0.19	0.34
C20:3n3	0.19	0.18	0.17	0.15	*0.02*	0.23	0.09	0.02	0.78	0.19
C20:5n3	0.05	0.03	0.05	0.06	*0.02*	0.99	0.48	0.67	0.35	0.64
C26:0	0.32	0.13	0.28	0.21	*0.07*	0.09	0.78	0.30	0.40	0.15
Denovo	35.7	34.0	34.9	31.9	*2.08*	0.28	0.50	0.37	0.76	0.86
Preformed	60.0	61.6	61.0	63.6	*1.97*	0.30	0.47	0.47	0.81	0.63
Bacteria	3.51	3.54	2.99	3.04	*0.09*	0.65	<.01	0.96	0.90	0.19
Δ9 C18[2]	0.64	0.61	0.65	0.66	*0.02*	0.68	0.23	0.91	0.40	0.85
Δ9 C16[2]	0.07	0.07	0.08	0.09	*0.01*	0.47	0.08	0.28	0.45	0.50
Δ9 C14[2]	0.05	0.06	0.04	0.05	*0.00*	0.22	<.01	0.03	0.96	0.57
Δ9[2]	0.35	0.34	0.36	0.39	*0.03*	0.69	0.27	0.42	0.52	0.92
Saturated	65.1	65.1	63.5	60.6	*2.5*	0.57	0.24	0.35	0.58	0.81
Unsaturated	34.6	34.6	35.9	38.6	*2.5*	0.60	0.30	0.42	0.59	0.98
PUFA	3.82	3.88	3.72	3.81	*0.20*	0.67	0.63	0.72	0.96	0.79

[2]Delta 9 desaturase indexes were calculated as follow:
Δ9 C14 = cis9 C14:1/(C14:0 + cis9 C14:1)
Δ9 C16 = cis9 C16:1/(C16:0 + cis9 C16:1)
Δ9 C18 = cis9 C18:1/(C18:0 + cis9 C18:1)
Δ9 C18:1 = cis9 trans12 C18:2/(trans12 C18:1 + cis9 trans12 C18:2)
Δ9 desaturation = sum of C14, C16, C18, trans12 C18:1 cis9/(sum of C14:0, C16:0, C18:0, trans12 C18:1 + sum of C14, C16, C18, trans12 C18:1 cis9)
